# The effect of age when group housed and other management factors on playing and non-nutritive sucking behaviour in dairy calves: a cross sectional observational study

**DOI:** 10.1186/s13028-020-00562-y

**Published:** 2020-11-19

**Authors:** Masja Reipurth, Stephanie Kruuse Klausen, Matthew Denwood, Björn Forkman, Hans Houe

**Affiliations:** 1 Department of Veterinary and Animal Sciences, Faculty of Health and Medical Sciences, University of Copenhagen, Groennegaardsvej 8, 1870 Frederiksberg C, Denmark; 2 Present Address: World Animal Protection Denmark, Amagertorv 29, 2nd floor, DK-1160 Copenhagen, Denmark

**Keywords:** Behavioural observations, Calf welfare, Social behaviour, Social housing

## Abstract

**Background:**

The aim of this study was to investigate if calves’ play behaviour and non-nutritive sucking behaviour, as indirect measures of welfare status, are associated with the age of the calf when group housed, age when observed, age difference within the group, pen size, milk feeding system, current or previous sicknesses, access to dry teat, indoor/outdoor rearing, sex, organic/conventional farm, group size and regrouping events. An observational study was conducted on 176 Danish dairy calves in the age range of 1–12 weeks, on both conventional (n = 17) and organic (n = 5) farms. All calves had been group housed before 8 weeks of age and had spent various periods of time with the dam and/or individually housed before being group housed. Behaviour was recorded continuously by filming each individual calf over a period of 30 min.

**Results:**

The calf’s age when group housed for the first time was not found to be significantly associated with duration of either play behaviour (P = 0.55) or non-nutritive sucking behaviour (P = 0.44). It was found that calves had significantly reduced odds of playing for longer than the mean play duration (5.5 s) for each day of their lives (OR = 0.97, P = 0.003). Also, they had reduced odds of performing non-nutritive sucking behaviour for longer than the mean non-nutritive sucking duration (145.5 s) when milk was allocated by drinker buckets fitted with a teat compared to by bowl or trough (OR = 0.06, P = 0.02).

**Conclusion:**

No significant associations were found between calves’ age when group housed for the first time and play and non-nutritive sucking behaviour. It was found that calves’ play behaviour decreased with increasing age, and that non-nutritive sucking behaviour decreased when milk was allocated with a teat compared to no teat.

## Background

In EU dairy production, calves are often separated from their dam shortly after birth, depending on the type of production [1, 2]. Subsequently, the calves can legally be housed individually, with only nose contact with other calves, until 8 weeks of age [3], unless raised within an organic system in which case the limit is 1 week of age [4]. Both the EU directive and Danish legislation acknowledge the importance of social contact between calves, but only state that calves *above* 8 weeks of age must be kept group housed, as opposed to immediately after birth. Farmers are allowed to group house their calves as early as desired, but due to a concern of disease spreading between the calves they are often kept in individual pens for as long as the legislation allows (8 weeks of age). The restricted social contact between calves has been criticized in terms of welfare for numerous reasons. Here amongst because non-domesticated calves have a strong social attraction to other calves from the age of 6 to 7 days, forming groups of up to 10 individuals in the wild [5], and because studies have shown that calves are more motivated to gain access to full contact rather than just nose contact with another calf [6]. Individual housing prevents calves from physical interaction such as play fighting with peers [7, 8], and has been found to be stressful because it offers no social buffering in a period of their lives during which they undergo separation from the dam, start using a drinking apparatus, and are weaned [9, 10]. Furthermore, the restricted social contact reduces or removes their possibility of learning social behaviours through interaction with other calves [6, 11].

Previous studies have examined behavioural, health and performance effects of the age of the calf when group housed for the first time. Some studies found no effects of age when group housed: 5-week-old calves that had been group housed 1 day or 3 weeks after separation from their dam had no differences in the social bonds between them [12]; 6-week-old calves that had been group housed 1 day or 2 weeks after separation from their dam showed no differences in fear responses [13], and calves from 3 days of age until 7 weeks of age that had been group housed at 3 days of age or 7 to 14 days of age showed no differences in health or performance [14]. However, the last study also found that the calves that were group housed early spent more time playing, but also performing cross sucking, than the calves that were group housed late [14].

Other studies found benefits of group housing calves early rather than late: 6-week-old calves that had been group housed at 5 days after separation from their dam had fewer vocalisations after milk weaning than calves group housed at 5 weeks of age [9]; pre-weaned and 10-weeks-old dairy calves’ intake of calf starter and daily weight gain was higher for calves that had been pair housed at 6 days of age than calves that had been pair housed at 6 weeks of age [15], and 7-week-old calves that had been group housed at 6 weeks of age had more difficulties learning reversal tasks and hence reduced abilities to respond to changing environments, than calves group housed at 6 days of age [16].

Lastly, one study found benefits of group housing late rather than early: Calves that were group housed at 6 days of age performed less licking, sniffing and more lying than calves that had been group housed at 14 days of age, which they point out could be an indication that introduction into a large group is intimidating for a very young calf, or because social interactions increase with increasing age.

The results of these studies are ambiguous when it comes to determining an optimal age for calves to be group housed. As illustrated in Table 1, 5 days, 6 days and 2 weeks have been found to have some sort of behavioural or performance benefit, compared to 5 weeks, 6 weeks and 6 days, while there were no benefits between 1 day, 2 weeks and 3 weeks, and both advantages and disadvantages for 1 to 2 days and 7 to 14 days. Hence, there it not yet a clear indication of which age is the most optimal to group house calves in order to achieve the highest level of welfare. Also, there are gaps in the representation of age groups in the studies, meaning not all ages have been examined. The reason for this is that the studies in Table 1 are experimental and compared fixed ages when group housed with one another, leaving out consideration of all other age stages from 0 to 8 weeks of age.

**Table 1:** Overview of studies on optimal age for group housing of dairy calves

Reference	Calves’ ages when tested	Age when group housed (A)	Age when group housed (B)
Abdelfattah et al. [14]	3 days to 7 weeks	*2 to 3 days*	*7 to 14 days*
Duve and Jensen [12]	5 weeks	*1 day*	*3 weeks*
Jensen and Larsen [13]	6 weeks	*1 day*	*2 weeks*
Bolt et al. [9]	6 weeks	***5 days***	5 weeks
Costa et al. [15]	4 to 10 weeks	***6 days***	6 weeks
Meagher et al. [16]	7 weeks	***6 days***	6 weeks
Rasmussen et al. [17]	1 to 4 weeks	6 days	***2 weeks***

The ages marked with bold italic indicates that this age had a benefit over the age without marking. The ages written with italics indicates no differences between the two ages, or that there are advantages and disadvantages for both ages

The aim of this study was to investigate if play behaviour and non-nutritive sucking were associated with the age at which the calves were group housed in order to examine whether calves experience a higher level of welfare if they are group housed before the legislative requirement of 8 weeks. The study is observational and includes all ages of calves, as well as all ages when group housed. Play behaviour was used as an indicator of positive welfare since it has been found to be associated with animals experiencing positive emotions [18, 19]. Non-nutritive sucking behaviour is caused by an unfulfilled motivation to suck the dam’s teat [20, 21]. The act itself can lead to ingestion of non-feed particles which has a direct effect on stomach upset (object-sucking) [22] and can result in health problems such as inflammation of the navel stump, hairlessness and increased infection risk in the suckled calf (cross-sucking) [23]. It is therefore used as an indicator of negative welfare.

The hypothesis was that duration of play behaviour would be positively associated, and non-nutritive sucking behaviour negatively associated, with early group housing of calves. Eleven other explanatory variables, including age difference in group, age when filmed, pen size, milk feeding system, current or previous sicknesses, access to dry teat, indoor/outdoor rearing, sex, organic/conventional farm, group size and regrouping events, were also included in the analysis.

## Methods

### Animals and housing

This study was carried out as an observational study between November 2017 and March 2018. The target population was commercial Danish dairy farms. The source population consisted of dairy farms on Zealand. The sample of farms was obtained by using a list from the Danish Central Herd Register (CHR) in which the Danish Veterinary and Food Administration register Danish herds and livestock [24]. The first 40 dairy farms on Zealand with more than 100 cows from this list were contacted; this minimum threshold for herd size was intended to result in farms with similar production levels. As the final and most essential inclusion criterion, only farms that grouped their calves together before 8 weeks of age (the threshold of the legislative demand in Denmark), and also had a group of calves in which all calves were equal to or younger than 12 weeks were included.

After telephone interviews with all 40 farm owners, a total of 30 out of the original 40 farms were found to meet these inclusion criteria. Of the 30, three would not participate, thus the total sampled population was 27 farms. The sampled population resulted in a total of 252 calves, divided into 51 calf groups, all of which were filmed. Of the 51 recordings, the quality of 13 of the groups, from five different farms, was too low to make observations, due to bad lighting, which meant they had to be discarded. Thus the final sampled population was 176 calves divided into 38 calf-groups (each consisting of between two and nine calves) on 22 different farms. All calves were 12 weeks old or younger and were grouped together with at least one other calf before 8 weeks of age.

### Data collection

We wanted to film the calves at the time of day when they performed the highest frequency of play and non-nutritive sucking behaviour. From previous studies it was found that calves’ performance of non-nutritive sucking (cross-sucking) peaked within the first 10 to 15 min after milk ingestion [21] and that play behaviour peaked around 9:00 to 11:00 in the morning, and again around 15:00 to 17:00 in the afternoon [5]. Therefor filming had to begin either after milk allocation in the morning or afternoon. Milk feeding times on the 22 different farms ranged from 04:30 h to 19:00 h. As the sun rises around 08:30 h and sets around 15:30 h in Denmark at the time of year where the study sampling was performed, most farms would allocate milk at least once a day while it was dark. It was decided to film the calves 60 min after milk had been ingested in the morning, as more farms had morning feedings after 08:30 h, than afternoon milk feedings before 15:30 h. On five of the farms, however, milk was allocated before sunrise; thus video recordings had to be postponed to the second feeding of the day when the light was sufficient. The video recordings were re-watched for each calf, and behaviour was recorded when the observed calf was no longer drinking milk, and therefore had pulled away from the milk feeding system. No interventions were implemented, and the observers left the barn at least 10 min before the filming began to avoid the calves acting differently in their presence. To obtain the video recordings, a GOPRO camera (Hero3) was used and placed to ensure the greatest full view of the calves and their pen. Using the recordings, the occurrence of specific behaviour was sampled for each individual calf (n = 176), operating a computer event recorder program called BORIS (version 5.1.3) [25]. Videos were recorded and viewed at normal speed. As it was observed that almost all calves laid down and slept or stayed inactive after approximately 25 – 30 min after milk ingestion, the behavioural recording time was reduced to the first 30 min out of the 60 min of observational recordings. During the behavioural observations, the two response variables, (1) play and (2) non-nutritive sucking, were assessed. Differentiations between the various categories of play and non-nutritive sucking behaviour, as described in Table 2, were not registered, as it was merely the behaviour itself that was the focus of the project, not the differentiations within the specific behaviour. Hence, the ethogram (Table 2) was simply used to identify the characteristics of the behaviours. The ethogram was developed based on previous published studies [18, 26]. The time at which play and non-nutritive sucking began and ended throughout the behaviour sampling period was recorded as a continuous measurement for each calf. The final total duration outcomes (response variable) were measured as how many seconds (s) out of the 30 min the behavioural performance had occurred. In addition to the two behaviours, a third registration, non-identifiable behaviour (NIB), was assessed. NIB included situations where the calf had its head out of the pen, or stood behind another calf so that its head movements could not be seen. The purpose of recording NIB duration was to ensure more accurate behavioural durations for non-nutritive sucking behaviour, as a calf could theoretically have performed this behaviour during NIB without it being registered. Therefore NIB duration was subtracted from the 30 min of observation for non-nutritive sucking behaviour when calculating percentage of time spent performing non-nutritive sucking behaviour. Play durations were not corrected for NIB duration, because play behaviour could always be observed even though the calf’s head was out of sight, due to large, easily-detectable movements. NIB durations are not presented in the results. Two observers with the same background in animal science carried out the assessments of the behaviour samplings. The total behaviour samplings were divided equally between the two observers.

**Table 2:** Ethogram for play and non-nutritive sucking behaviour

Observed behaviour	Description
Play	
Galloping/Running	Calf runs in circles, back and forth and/or in multiple and changeable directions
Bucking	Calf lifts both hind legs from the ground, resulting in a kick where both hind legs are stretched backwards in the air
Jumping and leap	Calf lifts both forelegs from the ground, and the hind legs may also be lifted from the ground at the end of the sequence
Turn	Calf suddenly turns in another direction, usually in a jump or running sequence
Head shake	Calf shakes or rotates its head
Frontal pushing or butting	Calf pushes the frontal part of its head against another calf’s head
Non-reproductive mounting	Calf lifts both forelegs to jump upon another calf’s back, side or head
Butting fixtures	Calf puts the front of its head against an object in the pen such as the bars (usually performed in a standing position)
Butting straw	Calf kneels in the straw and pushes the front of its head against the straw
Rubbing straw	Calf kneels in the straw and rubs its head, throat or neck down into the straw
Sucking	
Sucking on other individuals	Calf sucks on another calf’s ears, udder, foreskin, navel or other head- and body parts
Sucking on objects	Calf sucks on fixtures, including bars, buckets, teats, troughs and all other fixtures in the pen
Licking objects	Non-functional licking on fixtures, including bars, buckets, teats, troughs or any other fixture in the pen

The ethogram for play behaviour [18] is divided into 10 subcategories and non-nutritive sucking behaviour [26] is divided into three subcategories. No distinction was made between subcategories when observing and recording play and non-nutritive sucking behaviour

### Reliability

To ensure that the two observers had been calibrated adequately for assessing the calves’ behaviours, a Cohens’ Kappa weighted inter-rater agreement test was performed in R using RStudio (version 1.1.442). The test had two quantiles, with each behaviour divided into two using the mean duration as threshold. The data set used to estimate the level of agreement between the observers consisted of 29 observations (behaviour samplings), and accounted for seven calf-groups where both observers had recorded play and non-nutritive sucking occurrence in the same calves, but independently of each other. The measure of agreement (*K*) is adjusted by the agreement by chance, and the weight (*w*) ensures that the degree of disagreement is weighted differently dependent on the extent of the discrepancy [27].

### Explanatory variables

The explanatory variables were obtained through a number of different sources. Most were registered independently on the individual farm by the two observers (pen size, indoor/outdoor rearing, group size, milk feeding system and access to dry teat), while others were sampled by interviewing the farm owner of the specific farm (calf’s age when grouped, current or previous sicknesses, organic/conventional farm and regroupings). Regrouping was defined as any relocation of the calf after its first introduction to a group. Time of regrouping was not included in the analysis, as the farmers could only rarely remember exact times for the regrouping. How much time the calves spent with the dam prior to group housing could have been an interesting explanatory variable to include in the analysis. However, as with time of regrouping, exact time for when the individual calf was separated from the dam was not possible to extract. Grouping effects of farm and animal group were incorporated using anonymised farm/group labels in order to preserve the grouped structure of the data. The explanatory variables of age when filmed and sex of the calf were deduced using animal-level information from the CHR [24]. Finally, the explanatory variable for age difference in group was calculated by subtracting the age of the youngest from the oldest calf in the group.

The primary explanatory variable was the calf’s age when group housed for the first time, whereas the rest were secondary explanatory variables (see Table 3). All data measured for the response variables (play and non-nutritive sucking behaviour) were quantitative, and data gathered for the explanatory variables were both quantitative (n = 5) and qualitative (n = 7). Milk allocation method was originally divided into four categories; “trough”, “bowl”, “drinker bucket with teat” and “robot”, with “trough” as the reference value. However, the categories “robot” and “drinker bucket with teat” were considered to represent the same method of milk allocation and were therefore subsequently merged into a single category “drinker bucket with teat”, resulting in three categories in total. Troughs were large enough for all calves in the group to drink from the same trough, whereas bowls, drinker buckets with teats and robots had space for only one calf at the time.

**Table 3:** Overview of the explanatory variables (n = 12) included in the observational study

Population conditions	Explanatory variables Quantitative data = Q Qualitative data = q; q1 nominal and q2 dichotomised	Description
Individual level	Age when grouped (Q)	The decisive age for when calves are housed with other calves for the first time (days)
	Sex (q2)	Whether calves were heifer calves (h) or bull calves (b)
	Sickness (q2)	Whether any calf was sick or had been treated after birth (yes or no)
	Space per calf (Q)	The space in the pen per calf (m^2^)
	Age when filmed (Q)	The individual age of each calf in every calf-group (days)
Group level	Indoor- or outdoor rearing (q2)	If calves were placed indoors (i) or outdoors (o)
	Group size (Q)	The number of calves grouped together (number)
	Regrouping (q2)	Whether calves had been removed or released from the groups (yes or no)
	Conventional / organic farm (q2)	If the calf-group came from an organic farm (o) or a conventional farm (c)
	Age difference in group (Q)	The calculated age difference between the youngest and oldest calves in the group (days)
	Milk allocation method (q1)	What type of feeding system the calves get their milk allocated in: bowl, trough, robot, or drinker buckets with teat
	Accessibility to dry teats (q2)	Whether calves had dry teats in their pen (yes or no)

Of the 12 explanatory variables, five were recorded at the individual level and seven at the group level. Of the qualitative data (q), only milk allocation method was treated as nominal (q1), while six others were dichotomised (q2) for analyses

### Statistical analyses

The statistical analyses were performed using the lme4 package [28] for R, using RStudio (version 1.1.442). For each risk factor, a univariable mixed-effects logistic regression screening stage was conducted at first, one for each of the outcomes. Afterwards, two separate multivariable mixed-effects logistic regression models were performed, again with all the same variables, one for each of the outcomes. Some variables were significant at the univariable stage but were not significant within the multivariable model probably due to confounding with other variables. All outcomes (in seconds) for both response variables were dichotomised into high or low performance of behaviour with the same threshold values. The threshold was set as the mean duration, as we could not find any categorizations from previous studies of high and low performance of play and non-nutritive sucking behaviour that were relatable to and/or useable for this study. It was assumed that observations of calves within the same calf-group and observations of groups from the same farm were not independent, so random effects of farm (n = 22) and group identification number (n = 38) nested within farm were used in both models. The model equation for the probability P(x_i_) of the *ith* calf to be associated with an outcome above the threshold (play or non-nutritive sucking duration), given a value of the explanatory variable *x* for the *ith*calf*,* with random effects of farm and group identification number was:

$${\text{Logit }}\left( {{\text{P}}\left( {{\text{X}}_{{\text{i}}} } \right)} \right) \, = \, \alpha \, + {{\varvec{\upbeta}}}{\text{X}}_{{\text{i}}} + {\text{ u}}_{{{\text{Farm}}({\text{i}})}} + {\text{ u}}_{{{\text{Group}}({\text{i}})}}$$

where α is the intercept, *β* is the vector of coefficients, and X is the vector of predictors for calf *i.* The subscript *i* denotes the individual calf in the total sample size, *n*, of calves (*n* = 176). u_Farm (i)_ and u_Group (i)_ are referred to as the random farm- and group identification number effect, for the farm or group number of the *ith* calf.

Explanatory variables to be included in the multivariable analyses were selected using backwards elimination based on AIC [29], starting from a full model and testing all fixed-effect explanatory variables for exclusion. This involved comparing the AIC of the full model to the AIC of a series of models each excluding one of the explanatory variables, and selecting the model with the lowest AIC from this set as the updated model. This procedure was then repeated based on the updated model until no further improvement (i.e. reduction) in AIC could be obtained. The P-values, 95% confidence intervals (CI) and odds ratios (OR) associated with each explanatory variable were then noted from this final model. Within these models, OR can be interpreted as the odds ratio for calves to perform play or non-nutritive sucking behaviour with frequency below or above their respective threshold durations. The threshold for a significant result was set at P < 0.05.

## Results

### Reliability

The values of measure of agreement between the observers (*K*) for play and non-nutritive sucking behaviour were calculated to be 0.69 and 0.65, respectively, which can be interpreted as “good” agreement [27].

### Descriptive statistics

The age of the calves varied from 4 to 84 days (median = 52 days), and the age at which they were separated from their dam varied from 3 to 72 h (median = 24 h). After separation, most of the calves were housed individually before they were housed with other calves. The majority of the calves were group housed within the first week of life (n = 76), and the rest of the calves were group housed within 2 weeks (n = 35), 3 weeks (n = 27), 4 weeks (n = 36) and 5 and 7 weeks (both n = 1). How much time the calf spent with the dam and/or was individually housed before being group housed varied from 0 to approximately 49 days (median = 12 days), depending on various factors such as the calves’ individual health status or the individual farmer's standard farm procedures. The number of animals per group ranged from two to nine individuals (median = five individuals). Pen sizes varied from 1.4 to 9.6 m^2^ per calf (median = 2.4 m^2^). Feeding also varied for each calf group in relation to the time of feeding, the amount of milk allocated (between 5 and 12 L milk/day), and feeding systems used, which varied between trough, bowl, robot and drinker buckets with teat. No calves were given ad libitum access to milk. All calves had ad libitum access to various types of calf starter and hay, and all calves had access to water; most of them had ad libitum access from an automatic water cup, while the rest received water from manually filled water bowls or troughs, or from drinker buckets with a teat. Both sexes were represented, with 145 heifer- and 31 bull calves of various breeds; Holstein, Jersey, Red Danish Dairy cattle or mixed. Maximum, minimum, mean and median durations of play and non-nutritive sucking, measured in s of the total 30 min observation period, and total proportion in percentage of the accurate observation period (30 min minus total NIB duration in s) for all calves, (n = 176) are listed in Table 4.

**Table 4:** Real and proportional time spent performing play and non-nutritive sucking behaviour

	Max		Min		Mean		Median	
Play	310 s	17.2%	0 s	0%	21 s	1.5%	5 s	0.3%
Non-nutritive sucking	1353 s	79.5%	0 s	0%	223 s	16.3%	145 s	10.1%

Total duration in s of the total observational period, and total proportions, in percentage of the accurate observation period (which for non-nutritive sucking behaviour is excluded time spent performing NIB) for all calves (n = 176), for play and non-nutritive sucking

### Model results

**Fig. 1:**
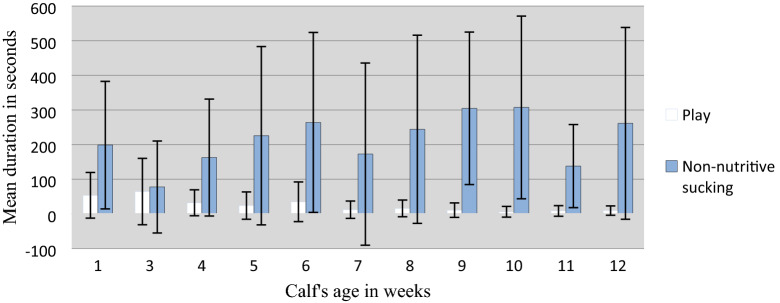
Mean duration of play and non-nutritive sucking behaviour plotted against the age of the calf when observed

**Fig. 2:**
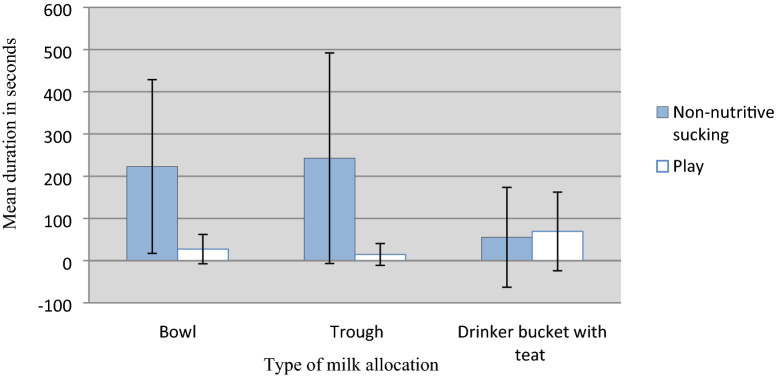
Mean duration of play and non-nutritive sucking behaviour plotted against milk feeding method: (1) Bowl, (2) Trough, or (3) Drinker bucket with teat

Results from the statistical analysis of the examined associations between play and non-nutritive sucking behaviour and the explanatory variables can be seen in Table 5. P-values, estimates, OR’s and 95% CI’s of the statistically significant explanatory variables throughout backward elimination can be seen in Table 6. Outcomes (duration in s of play and non-nutritive sucking) recorded during the behavioural observations (n = 176) for the statistically significant explanatory variables are illustrated in Figs. 1 and 2.

**Table 5:** P-values, estimates, OR’s and CI’s for all explanatory variables for play and non-nutritive sucking behaviour

	Play				Non-nutritive sucking			
	P-value	Estimate	OR	95% CI	P-value	Estimate	OR	95% CI
Age when grouped	0.72	− 0.009	0.99	[0.94; 1.05]	0.44	− 0.03	0.98	[0.92;1.05]
Age when filmed	*0.003*	− 0.04	0.97	[0.93; 0.99]	0.15	0.02	1.02	[0.99; 1.05]
Age difference in group	0.93	− 0.004	0.99	[0.95; 1.04]	0.65	0.009	1.009	[0.97; 1.06]
Group size	0.41	− 0.13	0.88	[0.63; 1.18]	0.13	0.24	1.27	[0.95; 1.73]
Space per calf	0.09	0.25	1.29	[0.97; 1.80]	0.72	0.07	1.07	[0.77; 1.50]
Sex	0.77	− 0.18	0.84	[0.28; 2.43]	0.93	0.05	1.05	[0.35; 3.23]
Sickness	0.19	− 0.84	0.43	[0.10; 1.34]	0.88	0.12	1.13	[0.24; 4.49]
Organic/conventional farm	0.50	0.37	0.69	[0.26; 2.02]	0.35	− 0.60	0.55	[0.12; 2.07]
Regrouping	0.06	− 0.97	0.38	[0.13; 1.09]	0.96	0.03	1.03	[0.31; 3.84]
Indoor / outdoor rearing	0.34	− 0.58	0.56	[0.16; 1.80]	0.79	0.18	1.19	[0.27; 4.42]
Dry teats	0.11	0.81	2.24	[0.89; 6.90]	0.27	− 0.68	0.51	[0.14; 1.46]
Milk allocation method								
Trough	–	0	1	–	–	0	1	–
Drinker bucket with teat	0.17	− 1.55	0.21	[0.04; 0.97]	*0.02*	− 2.90	0.06	[7.90e−18; 0.28]
Bowl	0.67	− 0.42	0.66	[0.10; 3.75]	0.57	0.45	1.57	[0.36; 11.46]

P-values, estimates, OR and 95% CI for OR from the logistic regression analyses of the association between the two response variables (play and non-nutritive sucking) and the 12 explanatory variables. Non-significant P-values, OR and CI are noted from immediately before the explanatory variable was dropped based on backwards elimination. Significant P-values are marked in italic. For milk allocation method, ‘Trough’ is the reference to which ‘Drinker bucket with teat’ and ‘bowl’ is compared

**Table 6:** P-values, estimates, OR’s and 95% CI’s of the explanatory variables that came out statistically significant (Age when filmed and Milk allocation: Drinker bucket with teat) for both outcomes (play and non-nutritive sucking) throughout backward elimination where 11 other explanatory variables where excluded from the multivariable logistic regression model

Drop no	P-value	Estimate	OR	95% CI	P-value	Estimate	OR	95% CI
	Play & Age when filmed				Non–nutritive sucking & Milk allocation: Drinker bucket with teat			
0	0.02	− 0.031	0.97	[0.94; 0.99]	0.37	− 1.374	0.25	[0.02; 1.9]
1	0.013	− 0.032	0.97	[0.94; 0.99]	0.36	− 1.398	0.25	[0.01; 2.12]
2	0.022	− 0.028	0.97	[0.95; 0.99]	0.37	− 1.349	0.26	[0.01; 1.91]
3	0.021	− 0.028	0.97	[0.95; 0.99]	0.39	− 1.253	0.29	[0.004; 2.36]
4	0.022	− 0.027	0.97	[0.95; 0.99]	0.33	− 1.376	0.26	[0.002; 1.79]
5	0.018	− 0.028	0.97	[0.95; 0.99]	0.29	− 1.479	0.23	[4.49e−07; 2.04]
6	0.017	− 0.029	0.97	[0.95; 0.99]	0.35	− 1.305	0.27	[5.24e−13; 2.39]
7	0.022	− 0.027	0.97	[0.95; 0.99]	0.27	− 1.517	0.22	[1.39e−37; 1.89]
8	0.024	− 0.026	0.97	[0.95; 0.99]	0.10	− 2.291	0.10	[2.85e−31; 0.81]
9	0.006	− 0.032	0.97	[0.95; 0.99]	0.06	− 2.564	0.08	[1.82e−33; 0.59]
10	0.008	− 0.032	0.97	[0.94; 0.99]	0.02	− 2.895	0.06	[7.90e−18; 0.28]
11	0.003	− 0.035	0.97	[0.93; 0.99]

According to Fig. 1, play behaviour was generally higher for younger than older calves, with a peak in performance at 3 weeks of age (64 s), and lowest for calves at 10 weeks of age (6 s). For the explanatory variable age when filmed, there was a significant negative association (P = 0.003) for the group of calves that played above the threshold duration (5.5 s), and an OR of 0.97. Hence, the odds of a calf playing above the threshold duration is reduced 0.97 times for every day, or in other words; calf play behaviour decreased by 1.2 times for every 1 week increase in calf age.

Throughout the backward elimination, the estimate for the variable Age when filmed for the outcome Play, varied between − 0.035 and − 0.026 (difference of 0.009 or 24%,) while OR did not vary and stayed at a solid 0.97 (Table 6). For non-nutritive sucking, the variable Drinker bucket with teat varied between − 2.895 and − 1.253 (difference of 1.642 or 57%) for the estimate, while OR varied between 0.06 and 0.29.

The explanatory variables age when grouped, age difference in group, regrouping, sickness, group size, sex, organic/conventional farm, indoor/outdoor rearing, space per calf, accessibility to dry teat and milk allocation method were not found to be significantly associated with play behaviour.

As can be seen in Fig. 2, non-nutritive sucking durations were on average higher when calves had their milk allocated by trough (243 s), compared to bowl (223 s) or drinker bucket with teat (55 s). The statistical analysis showed a significant negative association with milk allocation method, specifically the category “drinker bucket with teat” (P = 0.02), when the calves had non-nutritive sucking durations above the threshold duration (threshold duration = 145.5 s). The estimate for the category “drinker bucket with teat” resulted in an OR of 0.06, hence the odds for a calf that receives its milk in drinker buckets with teat to perform non-nutritive sucking behaviour above the threshold duration were almost 20 times lower than those in the reference category.

The remaining explanatory variables of age when grouped, age when filmed, age difference in group, group size, space per calf, sex, sickness, organic/conventional farm, regrouping, indoor/outdoor rearing and accessibility to dry teats were not found to be significantly associated with non-nutritive sucking behaviour.

## Discussion

### Play behaviour

One of the two main hypotheses in this study was that play duration would be positively associated with earlier grouping of calves. However, no significant association was found between play duration and grouping age in this study (P = 0.72).

It was found that there was a negative association between play behaviour and age when filmed. Calves had reduced odds of 0.97 to perform play behaviour above the threshold (5.5 s) for every day older they became older. This can also be seen in Fig. 1, where play behaviour shows a tendency to decrease from week 2. In other words, calves are a bit less likely to play the older they become, which is also consistent with results from other studies [18, 30, 31]. This could indicate that early rather than late grouping of calves might allow for them to perform larger quantities of play behaviour, but a previous study found that total duration of play behaviour did not increase with early rather than late grouping of calves. Only social play, which demands the presence of a peer, increased [32].

Due to more space per calf in the pens when calves are group housed compared to individually housed [26], group housing has been found to provide calves with better conditions for performing play behaviour [18]. However, in the current study, no associations were found between space per calf and play behaviour.

Frequency of head butting has previously been found to be higher for 2- to 6-week-old bull calves than heifer calves [33]. In the current study, there was no significant association between play and sex of the calf, and the behaviours were not divided into subcategories either.

Age when separated from the dam was originally a part of the interview of the farmer in the current study, but data had to be excluded as the estimates were too inexact. This could have been interesting data to include though, as is has previously been found that calves that had been reared by the dam for the first four days after birth and then group housed until 12 weeks of age had higher spontaneous play behaviour than calves of the same age that had been group housed immediately after birth [34]. However, there were no differences in total duration of play behaviour between the treatments in the study.

Many studies have found associations between energy intake and play. Here amongst that weight gain is positively correlated with frequency of locomotor play for 2- and 6-week-old heifer calves [33], and that low energy intake for calves 6 weeks of age is associated with reduced locomotor play [35]. In accordance with this, it has also been found that calves that got milk from an automatic milk feeder eight times a day initiated more social play than calves that got milk four times a day [17]. An explanation for this could be that more milk feedings makes calves stand up more, and calves standing are probably more motivated to play than calves lying in the hay [17]. In the current study, all calves received milk twice a day and this data was therefore not used as an explanatory variable.

Play behaviour was used as a measure of welfare in this study, since it has been found to be associated with animals experiencing positive emotions [18, 19, 31], and being healthy [33]. However, play behaviour as a welfare measure has also been criticized because the behaviour is performed too rarely to be able to draw any conclusions, and because of the uncertainty regarding whether play reflects a positive emotion or if calves use it as a coping strategy [36]. One study found that motivation for some, but not all, subcategories of play builds up over time, indicating that there are specific subcategories of play that are more suited as indicators of welfare than others [37]. Differences between social- and non-social play behaviour were not registered in the current study.

The sampling in this study was conducted during wintertime, which led to some complications in regard to daylight. On five of the 22 farms, recordings had to be postponed to the second milk feeding of the day, when light was sufficient. Previous studies found that calves had higher peaks of play between 09:00 h and 11:00 h in the morning [5], meaning that recordings on the five farms theoretically could take place outside peak time. Consequently, time of day for recording could be a source of bias resulting in outcomes with underestimated durations of play behaviour from the calves filmed in the evening. Another source of bias could be the behavioural video observations, as some of the calves’ movements could be misinterpreted as play behaviour. One example could be head shake, which in theory could be both a play behaviour, but also caused by en ear infection in the calf, or a sign of frustration [33]. This could lead to a small over- or underestimated recording of play behaviour.

To summarize, no association was found between play duration and age when first group housed. Play duration was found to be associated with age when filmed, indicating that calves play less the older they become. Since age when filmed was confounded with age at grouping no conclusion regarding the effect of treatment on play behaviour could be reached.

### Non-nutritive sucking behaviour

It was hypothesized that non-nutritive sucking behaviour would be negatively associated with earlier group housing of calves. In this study, no significant association was found between non-nutritive sucking duration and age when grouped. This is in accordance with Größbacher et al. [38] who found that age at grouping did not have a significant effect on cross-sucking for Simmental calves.

It was found that there was a negative association between non-nutritive sucking and feeding with Drinker bucket with teat. Calves had reduced odds of 0.06 to perform non-nutritive sucking behaviour above the threshold (145.5 s) when their milk was allocated by drinker buckets with teat compared to calves offered milk in bowls and troughs. In other words, calves performed less non-nutritive sucking behaviour when milk was allocated by teat. The difference is also seen in Fig. 2, where milk allocation that does not include teats results in non-nutritive sucking durations of 535% higher than milk allocation by teat (average of 87 s versus 465 s). This could indicate that milk ingested with the possibility to suck on a teat to a greater extent satisfies calves’ motivation to suck, compared to milk offered by bowl or trough (where milk is ingested without the possibility to suck on a teat). This is in agreement with previous studies who found significantly less non-nutritive sucking in calves that were offered milk by buckets fitted with a teat, compared to buckets without teats [39, 40], and that duration of sucking at buckets fitted with a teat was negatively correlated with the duration of cross-sucking [38]. Another study, however, found that there were no differences in cross-sucking between 8-week-old calves that had been allocated water with a nipple from the second day after birth until weaning, compared to water allocation without the possibility to suck on a nipple [41]. Hence, non-nutritive sucking appears to multifactorial, and not solely dependent on access to teat or nipple. Cross-sucking may be related to hunger [42, 43], and if this is assumed, then cross-sucking may be reduced by ensuring proper milk allowance to calves that are grouped together. Feeding with a teat with smaller tube diameter (1.5 mm compared to 3.0 and 6.0 mm) has also been found to decrease duration of non-nutritive sucking, indicating that the speed in which the calf receives milk by teat has an effect on non-nutritive sucking [43]. However, is has also been found that milk flow and portion size had no effect on occurrence of cross-sucking, but that bull calves performed and received more cross-sucking than heifer calves [44]. In this study, there were no significant differences between duration of non-nutritive sucking and calf’s gender.

In a previous study it was found that number of milk allocations had an influence on non-nutritive sucking. They found that calves that got milk allocated eight times a day from an automatic milk feeder sucked the empty teat more often than the calves that got milk four times a day [17], possibly because non-nutritive sucking is motivated by the taste of milk [21]. In the present study all calves received milk two times per day, hence number of milk allocations were not included as a response variable. Milk allowance and milk flow (due to various feeding systems) however, differed for each calf-group (n = 38). These variables were indirectly accounted for, as calf-groups and farms were included in the models as random effects.

Breed could have been a possible explanatory variable for non-nutritive sucking behaviour, as cross-sucking has been found to be more common among Jersey calves compared to other dairy breeds [45]. However, in this study, breed was not included as explanatory variable, as most of the calves were mixes of several different breeds.

To facilitate the assessments, the recorded behaviours were limited to two behaviours, with no differentiation of the behavioural sub-categories. Therefore, it is not possible to say how much of the behavioural performances were social or individual. For example, while both categories of sucking behaviour are non-nutritive, and are symptoms of a motivation to suck and/or sign of hunger [46], cross-sucking directly impacts the welfare of the receiving calf negatively because it can lead to, e.g., naval infection [23]. The specific category of non-nutritive sucking behaviour does probably not affect the welfare of the observed calf on an individual level. However, it would have been beneficial to be able to specify which explanatory variables had an influence on cross-sucking, as this category of non-nutritive sucking behaviour on a group level is associated with a higher decrease in welfare than object sucking.

To summarize, no association was found between non-nutritive sucking behaviour and age when grouped. It was found that non-nutritive sucking behaviour is associated with the “drinker bucket with a teat” feeding system, indicating that calves will perform less non-nutritive sucking behaviour if they are allocated milk from a bucket with a teat, compared to bowl or trough without teats.

### Welfare

While there are not too many studies examining the effects of calves’ age when group housed in relation to welfare, there are a number of studies on individual versus group housed calves using measures such as health, production, behaviour, etc., as indirect measures of welfare. Though age at introduction into a group is not included as an explanatory variable in any of the studies, the results are still of relevance as they can indicate in which environment the calf experiences the highest level of welfare, health, etc., and hence in which environment it makes sense to house the calves in until they legally have to be group housed at 8 weeks of age.

Amongst the negative effects in group housed calves compared with individually housed calves are increased incidence of diseases [13, 47, 48], reduced feed intake and weight gain [47] and higher mortality [49–51]. Others have found positive effects from providing social contact between pre-weaned dairy calves rather than housing them individually, such as higher total feed intake [32, 52, 53], higher weight gain and growth [32, 54, 55], stronger bonds with other calves [9, 11, 12], improved social confidence and development of normal social responses [56–58], higher activity level [53, 59], more play behaviour [18, 57, 59], decrease in restlessness [53], higher social ranks [11, 60, 61], lower anxiety/fear/stress responses [57, 62, 63], higher optimism [64] and better performance in cognitive tasks and earlier learning [65, 66].

Hence, the studies of individual versus group housing of calves seem to indicate that group housing generally has a negative effect on health, but a positive effect on performance, behaviour, cognitive benefits and welfare. Although it appears that the majority of studies indicate that group housed calves experience a higher level of welfare in some aspects, it cannot be logically deduced that earlier group housing of calves automatically increases the number of days with higher level of welfare for calves.

The aim of this study was partly to discover, whether calves had increased level of welfare when group housed early rather than late. From previous studies, it appears that group housed calves experience a higher level of welfare than individually housed calves. Lowering the age at which calves are group housed for the first time could therefore possibly increase the calves’ welfare, but the optimal age for grouping merits further research. In this study, no conclusions can be made as to whether calves have increased welfare when group housed earlier, based on performance of play behaviour and non-nutritive sucking. In the present study there was such a strong effect of age on play performance over even a comparatively short time period which makes it impossible to directly compare the play behaviour between the different ages of grouping. However, as cross sucking in previous studies has been associated with a decrease in welfare for the receiving calf, it is probable that milk allocation with the use of a teat increases welfare for the calf.

### Methods and statistical analyses

The measure of agreement (*K*) from the Cohens’ Kappa (*w*) test resulted in “good” agreements. The reason for the *K* not being higher for non-nutritive sucking behaviour is probably the fact that the performance of non-nutritive sucking behaviour could easily be misinterpreted as a performance of grooming behaviour where the calf, e.g., rubs another calf with its mouth. Play is a behaviour that is quite easily detectible due to its large movements. Logically, it should therefore result in a relatively larger *K* than for non-nutritive sucking behaviour, but this was not the case. Differences in recordings of the duration of the behaviour could be caused by the short pauses in between the different movements. These could both be interpreted as the end of a play session, or as a part of the play session where the calf takes a few seconds to calculate the next move.

In this study, twelve explanatory variables were used within the same multivariable logistic regression model in an effort to deal with confounding due to correlation between these explanatory variables. However, the correlation present between these explanatory variables may have resulted in elimination of biologically important variables during the backward elimination process. For example, the estimate for the variable Age when filmed (for the outcome play) varied between − 0.035 and − 0.026 during the backwards elimination process depending on the presence of other explanatory variables within the model (Table 6). The difference between the two estimates is 0.009, or 24%, which is large enough to consider the variable to be a confounder [27]. However, the direction of the association between play and Age when filmed is consistent, so although the difference between the estimates indicates that statistical confounding is present, we do not believe that this affects the biological conclusions made. Similarly, the estimate of the association between non-nutritive sucking and the variable Drinker bucket with teat varied between − 2.895 and − 1.253; i.e. a difference of 1.642 or 57%. Again, the difference between the estimates indicates that the variables are confounded [27]. In this case the direction of the effect is also consistent, although an OR varying between 0.06 and 0.29 suggests caution in interpreting the true strength of the association.

## Conclusions

Calves’ age when group housed was not associated with play or non-nutritive sucking behaviour in this study. Calves’ play behaviour decreases the older they become, and their non-nutritive sucking behaviour decreases when milk is allocated with a teat.

## Data Availability

The datasets used and/or analysed during the current study are available from the corresponding author on reasonable request.
